# Emergence of FRESH 3D printing as a platform for advanced tissue biofabrication

**DOI:** 10.1063/5.0032777

**Published:** 2021-02-16

**Authors:** Daniel J. Shiwarski, Andrew R. Hudson, Joshua W. Tashman, Adam W. Feinberg

**Affiliations:** 1 Department of Biomedical Engineering, Carnegie Mellon University, Pittsburgh, Pennsylvania 15213, USA; 2 Department of Materials Science and Engineering, Carnegie Mellon University, Pittsburgh, Pennsylvania 15213, USA

## Abstract

In tissue engineering, an unresolved challenge is how to build complex 3D scaffolds in order to recreate the structure and function of human tissues and organs. Additive manufacturing techniques, such as 3D bioprinting, have the potential to build biological material with unprecedented spatial control; however, printing soft biological materials in air often results in poor fidelity. Freeform Reversible Embedding of Suspended Hydrogels (FRESH) is an embedded printing approach that solves this problem by extruding bioinks within a yield-stress support bath that holds the bioinks in place until cured. In this Perspective, we discuss the challenges of 3D printing soft and liquid-like bioinks and the emergence for FRESH and related embedded printing techniques as a solution. This includes the development of FRESH and embedded 3D printing within the bioprinting field and the rapid growth in adoption, as well as the advantages of FRESH printing for biofabrication and the new research results this has enabled. Specific focus is on the customizability of the FRESH printing technique where the chemical composition of the yield-stress support bath and aqueous phase crosslinker can all be tailored for printing a wide range of bioinks in complex 3D structures. Finally, we look ahead at the future of FRESH printing, discussing both the challenges and the opportunities that we see as the biofabrication field develops.

## INTRODUCTION

Over the past decade, 3D bioprinting has transitioned from a niche research area into a biofabrication platform used to engineer a wide range of scaffolds and tissue constructs. There are many reasons for this growth, but a major one is the potential to precisely place biomaterials and cells in 3D space with the goal of recreating the structure and function of complex biological systems, from the cellular to organ scale. By far, the most popular 3D bioprinting approach is extrusion-based and uses a motion control platform based on fused deposition modeling (FDM) desktop-grade 3D printers. In general, a solution of biological material (bioink) consisting of extracellular matrix (ECM) proteins, cell suspensions, and/or organic hydrogels is loaded into a syringe pump extruder and deposited in a layer-by-layer manner to build the 3D object. However, a major obstacle to successful bioprinting is the distortion of the soft and liquid-like bioinks due to gravity and subsequent loss of print fidelity. Without physical support, most bioinks are challenging to print in a layer-by-layer manner and do not cure quickly enough and/or with sufficient rigidity to allow for structure stability during the printing process. Researchers have developed photo-crosslinkable, temperature-sensitive, and rheologically modified bioinks to counter the effects of gravity and enable 3D bioprinting in air.^1–4^ Yet, these approaches often require compromises in terms of the biological properties of the bioink in order to achieve the materials properties required for printability.^5–8^

**FIG. 1.**
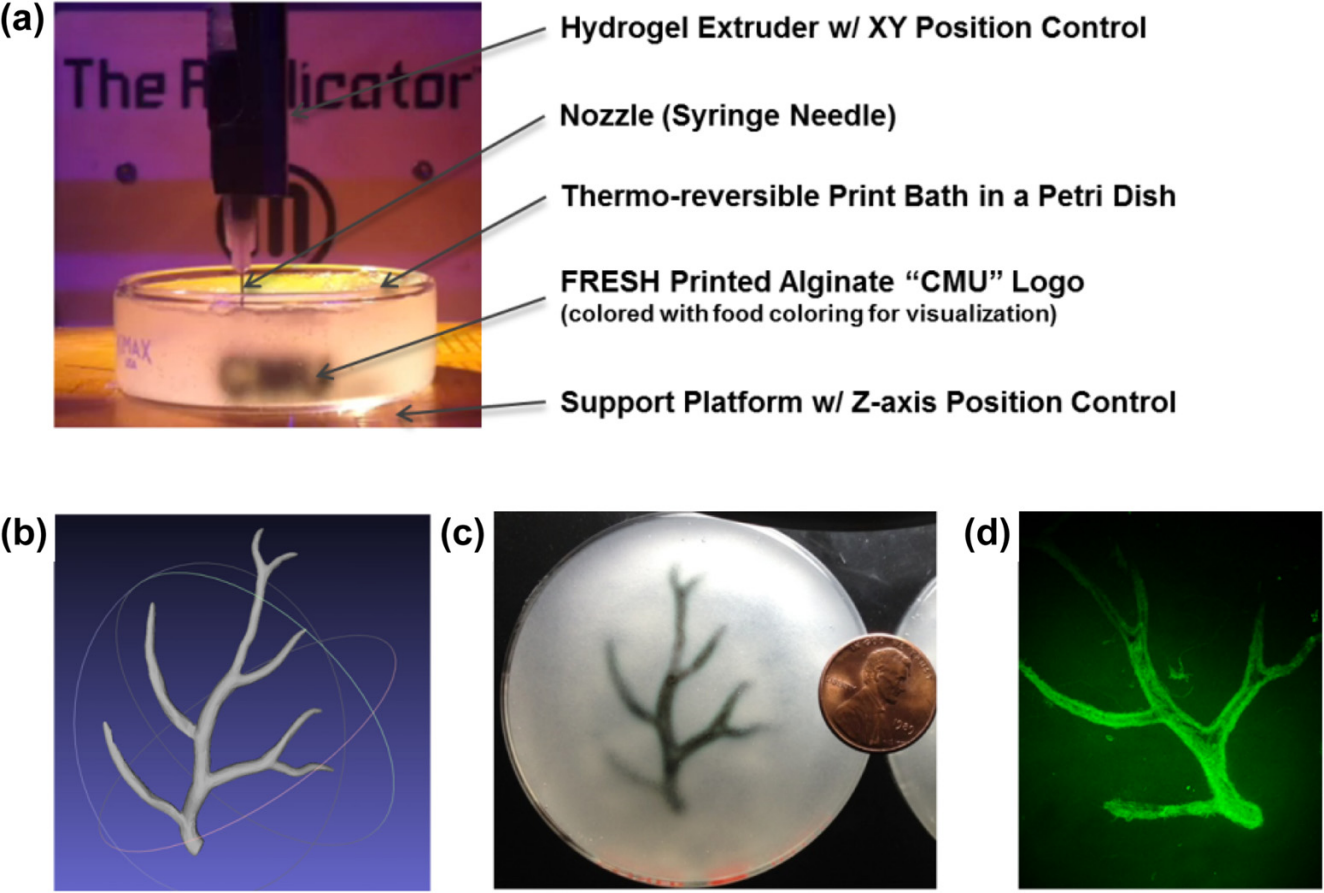
The FRESH printing approach. (a) FRESH 3D bioprinting consists of a syringe-based extruder that deposits biopolymers into a sacrificial support bath. In this example, the letters “CMU” (with black food coloring for visualization) are printed at a height of ∼5 mm. (b) A 3D computer-aided design (CAD) model of the coronary artery vasculature from a human heart to demonstrate the ability to print complex 3D structures. (c) A scaled-down FRESH print of the coronary artery vasculature using an alginate hydrogel (black) embedded within the support bath (gray) with a US penny for size reference. (d) The FRESH printed vasculature after release from the gelatin-based support bath and imaged using a confocal microscope shows the hollow lumen with a diameter of ∼1 mm and a resolution of ∼100 μm. Adapted with permission from Hinton *et al.*, Sci. Adv. **1**(9), e1500758 (2015). Copyright 2019 Authors, licensed under a Creative Commons Attribution (CC BY 4.0) license.^9^

Embedded 3D bioprinting has been developed as a solution to this challenge by providing a temporary and ubiquitous support structure during the printing process to decouple the bio ink gelation time and the cross-linking mechanism(s) from print fidelity. In general terms, one can think about embedded 3D bioprinting as a build chamber filled with a support material within which biomaterials, cell spheroids, cell-laden hydrogels, and other materials are deposited using a syringe-based extruder [Fig. 1(a)]. The key engineering challenge for embedded approaches is to develop a support bath material that can directly maintain the position of printed structures as they are extruded and cured while still allowing for the movement of the extruder needle through the support bath during printing. Thus, the support bath must possess a yield-stress behavior, such as Bingham plastic or Herschel–Bulkley fluid, where it acts as a solid until a sufficient shear stress (the yield stress) is applied, at which point it transitions from a solid to a liquid-like behavior. This phenomenon allows the syringe needle to be inserted into the support material and to traverse through it while extruding the bioink through the needle and into the liquid-like, yielded support. After the needle departs, the support resolidifies and locks the extruded material in place. This effectively embeds the bioink within the support bath, limiting the effects of gravity and holding the bioink in place until it cures.

Freeform Reversible Embedding of Suspended Hydrogels (FRESH) 3D bioprinting is an implementation of embedded printing developed specifically to overcome the limitations of printing soft and low viscosity bioinks.^9^ The goal is to be able to take complex 3D tissue and organ models [Fig. 1(b)], FRESH 3D print these models out of a wide range of biocompatible hydrogel and cell-laden bioinks within the support bath where the bioink will gel [Fig. 1(c)], and then release the printed construct [Fig. 1(d)]. In order to achieve this, FRESH embodies three unique aspects: (i) a support bath that acts as a viscoplastic material with Bingham plastic-like rheological behavior to achieve freeform fabrication, (ii) a customizable aqueous phase of the support bath compatible with the multiplexing of gelation mechanisms, and (iii) support bath liquification for nondestructive print release under biologically compatible conditions. Altogether, these features combine to create a printing environment that produces an unmatched combination of capabilities in terms of the materials that can be used (e.g., alginate, collagen, hyaluronic acid, fibrin, decellularized ECM, Matrigel, Pluronic® F-127, etc.),^9,10^ the resolution that can be achieved (∼20 *μ*m hydrogel filament diameter),^10^ and size and 3D geometric complexity that can be created.^9,10^ In this Perspective, we will explore the emergence of FRESH printing within the field of bioprinting and how embedded bioprinting techniques have transformed the landscape of tissue engineering by providing new capabilities for biofabrication. We will also address the current limitations of FRESH printing and discuss technological improvements and future applications of the platform.

## THE EMERGENCE OF FRESH AND EMBEDDED 3D PRINTING WITHIN THE BIOPRINTING FIELD

**FIG. 2.**
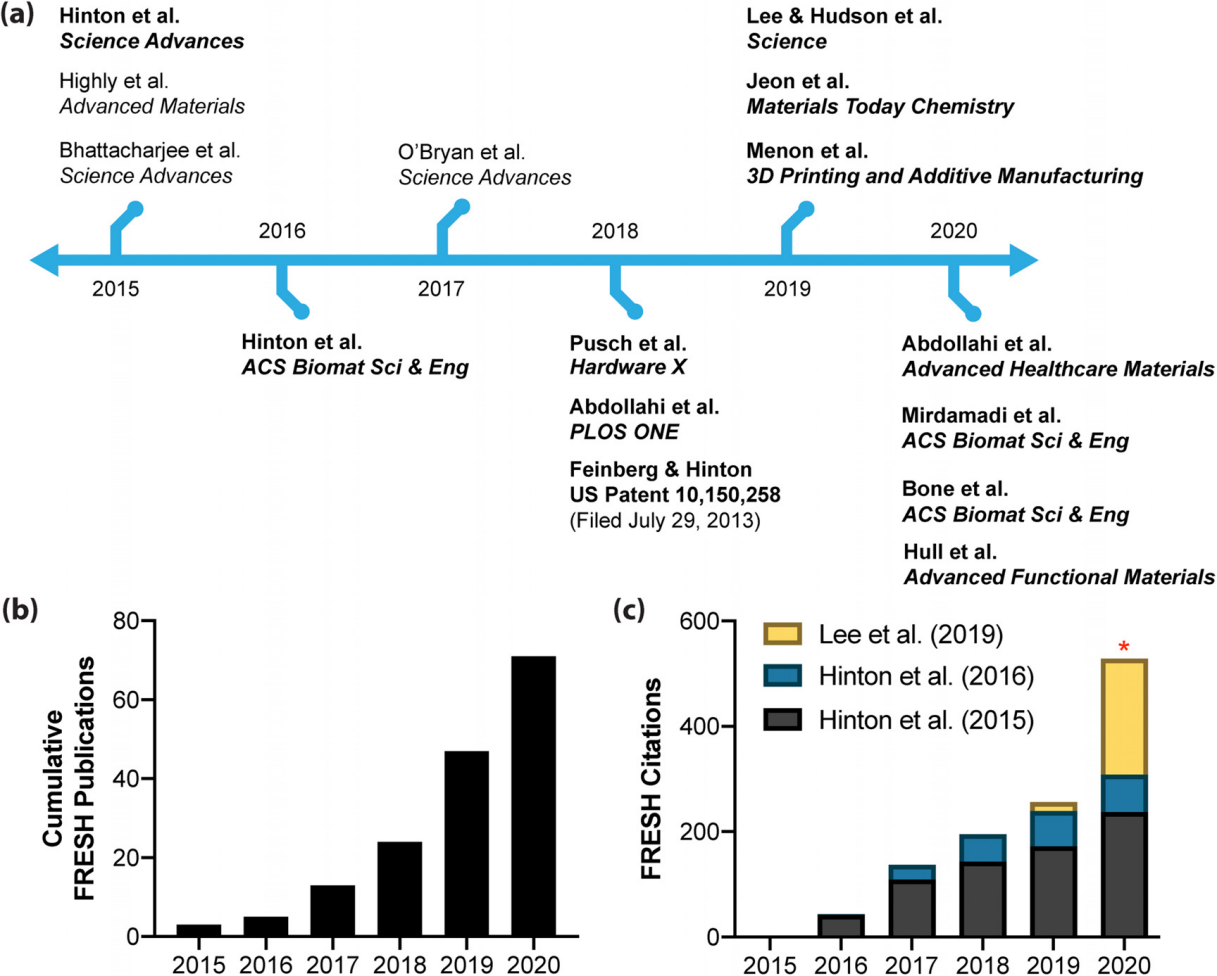
Timeline for FRESH and related embedded printing publications. (a) The timeline highlights key publications associated with the emergence of embedded printing into a yield-stress support bath in 2015,^9^ followed by subsequent publications on FRESH printing with a focus on those by Feinberg and co-workers (noted in bold text).^9–12,24–34^ (b) Publications using the FRESH printing platform or adapting the FRESH method since the initial publication by Hinton *et al.* in 2015.^9^ (c) Citations associated with three highly cited FRESH publications (Hinton *et al.* PMID-26601312;^9^ Hinton *et al.* PMID-27747289;^25^ Lee *et al.* PMID-3137612^10^). Asterisk denotes incomplete data for the current year.

In 2015, three seminal papers on embedded 3D printing within a yield-stress support bath were published within 3 months of each other in Advanced Materials and Science Advances, establishing the approach using different support bath chemistries [Fig. 2(a)].^9,11,12^ However, the need for support material to enable the 3D bioprinting of hydrogels and cells has been a long-established challenge in the field. For example, the Forgacs research group reported a cell spheroid printing method in 2006 using a simple collagen gel to hold the spheroids in position until they fused,^13^ followed by improvements where spheroids were placed within molds^14^ and within agar-based hydrogel layers.^15^ Because cell spheroids are solid-like and stable over the printing period, it is relatively straightforward to position them during bioprinting. However, hydrogel-based bioinks are quite different because they must be gelled or crosslinked, and this typically needs to happen *in situ* during the printing process. This need has been addressed in various ways depending on the bioink chemistry,^16^ including printing alginate in calcium chloride containing liquid baths for ionic cross-linking^17^ and UV irradiation of printed methacrylated gelatin for photocrosslinking.^18–21^ But hydrogel-based bioinks are also mechanically weak, and after gelation they need physical support to maintain their shape, especially when fabricating more complex 3D structures with overhangs. Approaches have largely focused on printing a sacrificial material such as Pluronic F-127 that can be removed after printing,^22,23^ but the geometric complexity is still limited. In 2011, Wu and co-workers were one of the first to utilize an embedded 3D printing approach where Pluronic F-127 was printed in a sacrificial support material composed of a UV curable Pluronic F-127 diacrylate matrix.^22^ The omni-directional printing that could be achieved in the support bath enabled fabrication of a bioinspired 3D microvascular structure and demonstrated the potential of using a support bath to build more complex scaffolds. At this time, Feinberg and co-workers were already developing the FRESH 3D bioprinting approach, experimenting with yield-stress support baths with Bingham plastic rheological properties. It was realized that the material properties of biological hydrogels presented several major challenges for a 3D layer-by-layer additive manufacturing process and that a method to decrease the effects of gravity by supporting a range of hydrogel gelation mechanisms during the printing process was needed.

When forming the concept that would become FRESH 3D printing in the study by Hinton *et al.*,^9^ we were drawn to the idea in science fiction of a patient being treated for injuries while floating in a rejuvenation tank. The phenomenon that allows for this suspension, neutral buoyancy, seemed to be a possible solution; however, numerous problems would arise when trying to print hydrogels into miscible water-based support fluids. Instead, the idea evolved to minimize the effects of gravity by using a gel-like support material that could reversibly transition from solid to liquid-like with material properties that would still allow for hydrogel deposition within it. FRESH was conceived of and developed specifically to achieve this capability and enable the 3D printing of a broad range of biologic and synthetic materials. Historically, the provisional patent covering FRESH printing and other embedded printing approaches within a yield-stress support bath by Hinton and Feinberg was filed in 2013 and granted in 2018 under U.S. patent number 10,150,258.^24^ This was followed in 2015 by the three seminal papers on embedded 3D printing using a yield-stress support bath by the Burdick, Angelini, and Feinberg laboratories.^9,11,12^ The goal of FRESH was to be able to 3D print unmodified biological hydrogels such as collagen type I and decellularized ECM with high density cell-laden bioinks for fabrication of functional engineered tissues. Specifically, the key aspects of FRESH are (1) a yield-stress support bath, (2) an aqueous phase compatible with multiple gelation mechanisms, and (3) controlled support bath liquification for nondestructive print release. Altogether, these features combine to create a freeform-embedded printing environment that produces unique printing capabilities with widespread advantages in biofabrication.

Since its initial development and publication in 2015,^9^ FRESH printing of soft and low viscosity bioinks in an embedded environment has seen significant growth within the field. Feinberg and co-workers have greater than 10 publications on FRESH and the related Freeform Reversible Embedding (FRE) printing process, where FRE is simply a more general term for FRESH where the ink is not a hydrogel. This includes printing of functional and cellularized tissue constructs,^10,29^ printing of Polydimethylsiloxane (PDMS) and medical devices,^25,28,31^ optimization and machine learning approaches to improve print fidelity,^28,30,33^ and expanding the size of FRESH-printed scaffolds to the organ scale.^27,32^ The Angelini research group similarly have multiple publications, focusing on the physics of the embedded printing process,^35,36^ new materials for the support bath,^26,37^ and cell behavior in 3D environments.^38–40^ Daly and co-workers have also explored 3D printing in yield-stress support baths using a range of chemistries and hydrogel microparticles.^41^ The impact of FRESH printing is also apparent by the large number of research labs that have adopted the technique. A few examples include the FRESH printing of nanocellulose,^42^ conductive hydrogels,^43^ scaffolds for stem cell growth,^44^ and ventricle-like heart chambers composed of beating cardiomyocytes.^45^ Overall, FRESH printing has become rapidly adopted in a growing number of publications [Fig. 2(b)] and key FRESH papers have been highly cited [Fig. 2(c)]. It is expected that FRESH will continue to be adopted now that FluidForm Inc. manufactures a research-grade FRESH support bath called LifeSupport^TM^ that is distributed by major bioink and bioprinter companies including Advanced Biomatrix, CELLINK, and Allevi. Finally, a number of researchers have effectively adopted FRESH printing by making small changes to the process. This includes using agarose-based support baths termed Suspended Layer Additive Manufacturing (SLAM)^46^ and Constructs Laid in Agarose Slurry Suspension (CLASS),^47^ and alginate-based support baths.^48–50^ While these publications and acronyms suggest a new technique, these are in effect FRESH printing and these alternatives to the gelatin support bath were described in the patent application by Feinberg and Hinton, published in February 2015 and granted in December 2018.^24^ While there is a lack of standardization in the 3D bioprinting field, the growth of FRESH printing and related embedded techniques is clearly moving in that direction, due in large part to the noted advantages of the approach.

## THE ADVANTAGES OF FRESH AND EMBEDDED 3D PRINTING FOR BIOFABRICATION

**FIG. 3.**
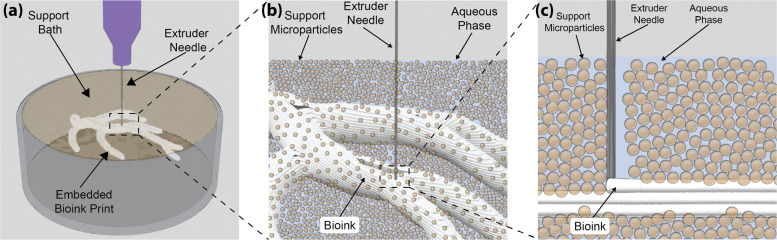
FRESH printing into a gelatin microparticle support bath. (a) A print container is filled with a yield-stress gelatin microparticle support bath to serve as the embedding medium for bioprinted components. (b) The aqueous phase of the support bath can be tuned to drive cross-linking/gelation of the extruded hydrogel as the microparticles support the print during layer-by-layer deposition. (c) As the needle moves through the support bath, the microparticles yield providing a space for the extruded bioink and subsequently heal behind the needle providing support to the curing bioink.

A key advantage of embedded approaches is the use of a support bath that can maintain the position of printed structures as they cure while still allowing for the movement of the extrusion needle [Fig. 3(a)]. In both the studies by Hinton *et al.*^9^ and Bhattacharjee *et al.*,^11^ this was achieved with jammed microparticles, as such systems manifest yield-stress behavior. Specifically, the support bath acts as a solid below a threshold applied shear stress (i.e., the yield stress) and then transitions from solid to liquid-like behavior above this threshold. This phenomenon allows a syringe needle to be inserted into the support bath and to traverse through it with ease during the printing process. Around the needle, the support bath liquifies because it exceeds the yield stress, and when the needle departs, the shear stress drops and the support bath resolidifies. During this process, a bioink or other material is extruded through the needle and displaces the liquified support bath. After the needle departs, the support bath resolidifies and immobilizes the extruded material in place. This effectively eliminates the effects of gravity on the extruded filament by supporting it on all sides [Fig. 3(b)].

The support bath in FRESH is composed of hydrogel microparticles and a surrounding aqueous phase, both of which are important for the 3D bioprinting process [Fig. 3(c)]. Hinton *et al.* focused on gelatin microparticles for the biocompatibility, low cost, and thermoreversible properties.^9^ Initially, a solid block of gelatin was blended to form microparticles with an average diameter of ∼60 *μ*m. However, this resulted in a wide range of sizes that negatively impacted the morphology of printed filaments. Lee *et al.* improved on this by using coacervation to produce finer, relatively monodisperse gelatin microparticles.^10^ The advantage of the gelatin microparticles is that they are stable at room temperature during the printing process, then can be melted at 37 °C after printing, and washed out to release the print. This is a process that is compatible with cells and proteins and is very gentle, meaning that even extremely soft scaffolds can be printed successfully and then removed.

The microparticles are typically mixed with an aqueous buffer compatible with cell culture and/or may contain a cross-linking agent, and then centrifuged to compact the microparticles into the aforementioned jammed state [Fig. 3(c)]. For example, to print an alginate bioink using FRESH, a solution of CaCl_2_ is added to the liquid phase of the microparticle support bath during its preparation and a 4% w/v alginate bioink solution is prepared in a syringe. As the alginate is extruded into the microparticle support bath containing CaCl_2_, the alginate is rapidly crosslinked. This process allows for an indefinite bioink pot life and extended printing times because the hydrogel will only cure once it is extruded into the support bath. Additionally, the rapid cross-linking helps to stabilize the printed structure, preventing unwanted deformations and diffusion during the printing process. Similarly, hydrogels, such as collagen and decellularized ECM, are easily FRESH printed by incorporating a pH buffer into the support bath aqueous phase such as HEPES. As acidified collagen or decellularized and solubilized ECM is extruded into the buffered support bath, rapid pH neutralization occurs in the vicinity of the extruded material driving the initial gelation process. The same approach can be extended to any catalyst-driven hydrogel with solubility in an aqueous buffer such as phosphate buffered saline (PBS) and has broad applicability across a range of gelation mechanisms (Table I).

**TABLE I.** Examples of bioink, aqueous phase crosslinker, and gelation mechanism for FRESH printing and commonly used bioinks and their appropriate crosslinker that can be incorporated into the aqueous phase of the FRESH microparticle support bath.

Bioink	Crosslinker	Gelation mechanism
Alginate	CaCl_2_	Ionic
Fibrinogen	Thrombin	Enzymatic
Collagen type I (acidified)	pH buffer (e.g., PBS, NaOH, and 50 mM HEPES)	pH change
Decellularized ECM (acidified)	pH buffer (e.g., PBS, NaOH, and 50 mM HEPES)	pH change
Methacrylated gelatin	Photoinitiator (e.g., Irgacure, LAP, and Riboflavin)	Photocrosslinking
Methacrylated hyaluronic acid	Photoinitiator (e.g., Irgacure, LAP, and Riboflavin)	Photocrosslinking

The FRESH support bath also provides an environment during the printing process that prevents cell death and maintains high cell viability. Since the aqueous phase of the gelatin microparticle support bath can be tuned to meet specific experimental requirements, pH neutral cell culture media can be used as the base for the aqueous phase to provide nutrients and oxygen to the cells during printing. This is in distinct contrast to a 3D bioprinter that extrudes cell-laden hydrogel bioinks in air because these immediately start to dehydrate and negatively impact cell viability.^51^ Additionally, a buffer solution such as HEPES can be added to allow cell survival in a CO_2_ independent environment and to neutralize printed acidified bioinks such as collagen type I and decellularized ECM. Furthermore, additional growth factors or biomolecules can be supplemented into the support bath aqueous phase to aid in cell survival as needed. We have previously shown that high density cell bioinks maintain >90% cell viability following FRESH printing and that printed human cardiomyocytes can assemble into functionally contractile electromechanically coupled tissues.^10^ Future effort to improve cell compatibility could allow for environmental temperature control of cell-based bioinks to control their basal metabolic rate and extend the window of printability as well as improving printing speed to limit the time that cells spend at room temperature outside of an incubator.

## CUSTOMIZABILITY OF THE FRESH PLATFORM

**FIG. 4.**
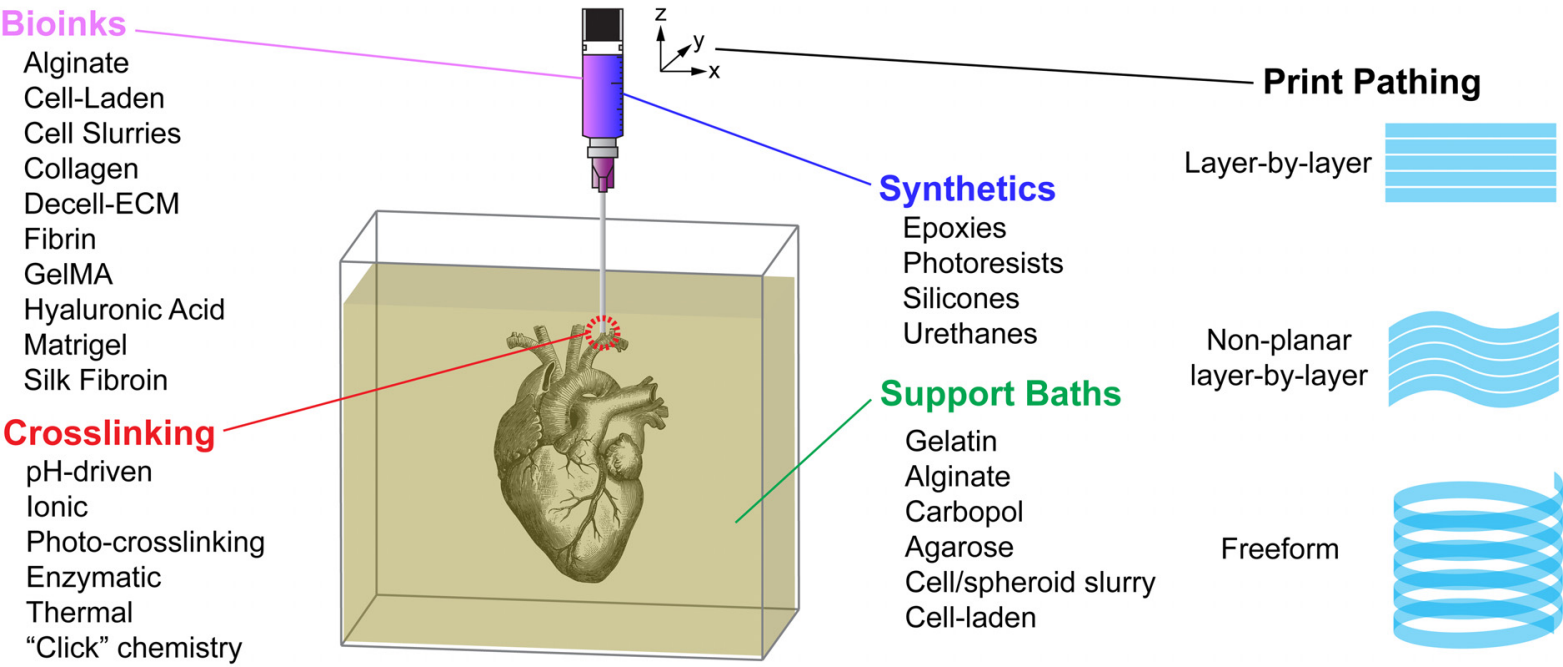
Customizability of the FRESH bioprinting platform. The FRESH platform allows for the bioink, support bath material, and aqueous phase to be tuned to utilize a diverse range of both biological and synthetic inks. Additionally, the embedded nature of FRESH printing provides capabilities to print using layer-by-layer, nonplaner, or freeform print pathing to produce geometries that are not possible in traditional bioprinting.

FRESH offers a wide range of customizability by changing the microparticle support bath to meet the needs of various bioink cross-linking mechanisms, biological or synthetic fabrication needs, and print pathing options. Although FRESH was originally described using a gelatin-based support bath, it can encompass a wide range of support bath materials. This is due to how FRESH support baths are defined primarily by materials having a yield stress rather than a specific chemical makeup. A simple but effective strategy to produce a yield stress material is to compact a slurry containing gel microparticles surrounded by a fluid phase. Compacting particles via centrifugation drives interstitial fluid out from between the particles, resulting in a jammed state. These jammed particles with minimal interfacial fluid are what can lead to a bulk yield stress material. This approach has been shown to work with numerous materials such as gelatin,^9,10^ alginate,^49^ agarose,^47^ and even cell spheroids.^52^ Additionally, several other research groups have modified the FRESH approach to create support baths from gellan gum,^53,54^ laponite nanoclay,^53,55–58^ and acrylamide.^38^ A range of example support baths are shown in Fig. 4 and listed in Table II. Each of these approaches has unique characteristics but shares the fundamental principles of utilizing a yield stress material with Bingham plastic or Herschel Bulkley rheological behavior similar to the original gelatin microparticle support.

**TABLE II.** Examples of FRESH and embedded printing support baths and inks. This table shows a range of biological and synthetic embedded 3D printing applications that highlight the versatility and customizability of FRESH and embedded printing. Abbreviations used in the table are as follows: carbon nanotubes (CNT), poly(3,4-ethylenedioxythiophene) polystyrene sulfonate (PEDOT:PSS), bicyclo[6.1.0]nonyne (BCN), arginylglycylaspartic acid (RGD), and popolyacrylic acid (PAA).

Bath material	Ink material	Lead author	Biological/synthetic	Use case
Acrylamide	Collagen-I cell mixture	Morley *et al.*^38^	Biological	Cell generated forces in bioprinted constructs
Agarose	Laponite–gellan gum	Cidonio *et a*l.^53^	Biological	Bone constructs
	Gellan/hydroxyapatite	Moxon *et al.*^83^	Biological	Osteochondral bioprinting
	Nanocomposite bioink alginate/collagen	Mendes *et al.*^80^	Biological	Hierarchical fibrillar structures
		Mirdamadi *et al.*^47^	Biological	Cell-laden hydrogel constructs
	Collagen, gelatin, and alginate			
		Senior *et al.*^46^	Biological	Hydrogel constructs
Alginate	Cell ink	Jeon *et al.*^29^	Biological	Tissue constructs
Alginate and xanthan gum	Cardiomyocyte omentum gel	Noor *et al.*^49^	Biological	Cardiac tissue constructs
Carbopol	Alginate/GelMA	Krishnamoorthy *et al.*^75^	Biological	Dual network cellular constructs
	Polyvinyl alcohol	O'Bryan *et al.*^26^	Synthetic	Support material development
	Bioelastomer prepolymers	Savoji *et al.*^89^	Biological	Vascular tubes
	PDMS 184	Bhattacharjee *et al.*^11^	Biological	Bioprinting technique development
	Silicone oil	Zhao *et al.*^60^	Synthetic	Silicone printing
	PDMS 184	Abdollahi *et al.*^28^	Synthetic	Synthetic printing optimization
	PDMS 184	Hinton *et al.*^25^	Synthetic	Silicone printing
	PDMS 184	Menon *et al.*^30^	Synthetic	Machine learning optimization of silicone printing
	Urethane and epoxy	Hajash *et al.*^59^	Synthetic	Large-scale synthetics printing
	Liquid metal	Yu *et al.*^95^	Synthetic	Liquid metal printing
	GelMA/gelatin/tropoelastin	Lee *et al.*^96^	Biological	Bioprinting of elastin containing bioink
	Cell sheroids	Ayan *et al.*^63^	Biological	Aspiration-assisted bioprinting of tissue spheroids
Cell spheroids or organoids	Cell spheroids and organoids	Skylar-Scott *et al.*^52^	Biological	High cellular density tissue constructs
EcoFlex elastomer	Carbon conductive grease	Muth *et al.*^84^	Synthetic	Strain sensor printing
Fumed silica	PDMS 184	Jin *et al.*^73^	Synthetic	Hydrophobic support bath
Gelatin	Alginate/collagen	Bessler *et al.*^66^	Biological	Printing hardware development
	Cellulose, alginate, and CNT	Bordoni *et al.*^42^	Biological	Neuroblastoma differentiation
	Decellularized vascular ECM	Choi *et al.*^68^	Biological	Muscle tissue constructs
	Alginate and collagen	Hinton *et al.*^9^	Biological	Bioprinting development
	Alginate and collagen	Isaacson *et al.*^72^	Biological	Corneal stroma printing
	Alginate	Jeon *et al.*^29^	Biological	Tissue constructs
	Collagen, alginate, MeHA, fibrinogen, and cells	Lee *et al.*^10^	Biological	Collagen and cell printing for cardiac tissues
	Alginate	Lewicki *et al.*^76^	Biological	Neuroblastoma printing
	Alginate	Lindsay *et al.*^44^	Biological	Stem cell expansion
	HA/collagen	Maloney *et al.*^78^	Biological	Chemotherapy screening
	Collagen	Maxson *et al.*^79^	Biological	*In vivo* remodeling of bioprinted constructs
	Hydroxyapatite/collagen	Montalbano *et al.*^82^	Biological	Bone scaffolds
	Cell laden collagen	Noble *et al.*^85^	Biological	Mechanics of recellularized scaffolds
	Alginate/callus/cells	Park *et al.*^86^	Biological	Food printing
	Alginate	Pusch *et al.*^27^	Biological	Large volume printing hardware
	GelMA and PEDOT:PSS	Spencer *et al.*^43^	Biological	Tunable ink mechanics
	Xanthan gum	Štumberger and Vihar^93^	Biological	Microfluidics
	Alginate	Wang and Florczyk^94^	Biological	Hierarchical porosity scaffolds
	Alginate and MeHA	Chen *et al.*^67^	Biological	Two-step crosslinked nanocomposite scaffolds
	MITCH-alginate	Dubbin *et al.*^51^	Biological	Dual-stage crosslinking for improved cell laden inks
	Alginate	Mirdamadi *et al.*^32^	Biological	Bioprinting tissue phantoms for surgical planning
	Union inks	Hull *et al.*^34^	Biological	Universal orthogonal bioink development
	Alginate	Shen and McCloskey^90^	Biological	Embedded concentration gradients
	Collagen	Montalbano *et al.*^81^	Biological	Nanostructured bone scaffolds
	Alginate	Bone *et al.*^33^	Biological	Machine learning to improve print fidelity
	Alginate/nanocellulose	Bordoni *et al.*^42^	Biological	Printing and differentiation of neuroblastoma cells
	Chitosan	Bao *et al.*^64^	Biological	Triggered micropore-forming bioprinting
	Hyaluronic acid (BCN-modified)	Aronsson *et al.*^62^	Biological	Peptide biofunctionalization of bioinks
	GelMA	Kupfer *et al.*^45^	Biological	Cardiac chambered organoid development
Gellan gum	Gelatin and alginate	Compaan *et al.*^54^	Biological	Support material development
Hyaluronic acid (HA)	Hyaluronic acid–RGD	Shi *et al.*^91^	Biological	Ink and support material development
	Hyaluronic acid	Highley *et al.*^12^	Biological	Ink and support material development
	Hyaluronic acid	Song *et al.*^92^	Biological	Endothelialized microchannels
	NorHA and PEGDA microgels	Highley *et al.*^71^	Biological	Microgel ink development
Laponite nanoclay	Alginate/gelatin	Jin *et al.*^57^	Biological	Support material development
	Alginate	Jin *et al.*^56^	Synthetic	Support material development
	Silk fibroin	Rodriguez *et al.*	Biological	Silk printing
Methanol, ethanol	PDMS 184	Karyappa *et al.*^74^	Synthetic	Silicone printing
Nanoclay	Gelatin and alginate	Ding and Chang^69^	Biological	Tubular structures
Oil gel bath	PDMS 184	O'Bryan *et al.*^26^	Synthetic	Silicone printing development
PDMS 1700	Pluronic F127	Grosskopf *et al.*^70^	Synthetic	Fluid mechanics in embedded printing
Pluronic F-127	Alginate	Rocca *et al.*^88^	Biological	Multi-ink printing
	Pluronic F127-acrylate	Wu *et al.*^22^	Biological	Microvascular networks
	Pluronic F127-dimethyacrylate	Basu *et al.*^65^	Synthetic	Catalytically activated polymerization
	Alginate	Afghah *et al.*^61^	Biological	Laponite pluronic support bath development
Poly(ethylene oxide) (PEO)	PAA–dextran (DEX) inks	Luo *et al.*^77^	Biological	Ink and support material development
Xanthan gum methacrylate	Alginate	Patrício *et al.*^87^	Biological	Ink and support material development

While FRESH printing can be performed with a wide range of support baths based on different materials, the customizability of FRESH ultimately lies in the variety of bioinks that can be printed. Specifically, FRESH enables the printing of soft materials with the broadest range of gelation mechanisms of any current bioprinting approach by leveraging the bioink–bath interface during printing. Upon being extruded into the bath, the bioink is embedded and exposed to the bath environment while being mechanically stabilized. Mixing cross-linking agents into the bath allows the extruded bioink filament to be immediately exposed on all sides, rapidly initiating the gelation process. This is in distinct contrast to printing in air, where there are limited ways to initiate gelation at the bioink–air interface. Gelation mechanisms include pH buffers, enzymes, ions, cell media, and photoinitiators, which allow for the printing of collagen, fibrin, alginate, cellular, and UV-sensitive bioinks [gelatin methacrylate (GelMA), methacrylated hyaluronic acid (MeHA), and poly(ethylene glycol) diacrylate (PEGDA), etc.], respectively. In addition to bioinks and biomaterials for tissue engineering and regenerative medicine applications, the FRESH platform also enables the use of a much broader range of synthetic polymer inks such as epoxies,^59^ silicones,^60^ urethanes,^59^ and photoresist^57^ when paired with the appropriate support bath. A range of biological and synthetic inks paired with various support baths and compatible cross-linking chemistry is shown in Fig. 4 and listed in Table II.^6,61–96^

The fluid phase of the support bath is as easy to interchange as the particulate phase; however, some chemical incompatibilities prevent some combinations of baths, bioinks, and cross-linking chemistries from being used together. Most notably, in order for cross-linking chemistries for multiple bioinks to be independent, they must be “orthogonal” or noninteractive with both the support bath and other bioinks. For example, separate collagen, alginate, fibrinogen, and MeHA bioinks can be FRESH printed into the same support bath by incorporating a pH buffer, calcium ions, thrombin, and photoinitiator, respectively. The printing of high-density cell bioinks (>200 million cell/ml) is also possible by incorporating cell media into the fluid phase, producing printed tissues that are far closer to *in vivo* cellularity.^10^ There are of course limitations and bioinks that require conflicting gelation conditions that are not compatible, such as an acidic bioink requiring a basic bath and a basic bioink requiring an acidic bath that cannot be printed together as the resulting baths, when mixed together, produce a neutral bath that cannot cross-link either bioink. The support bath must also be considered because a crosslinker that would cross-link both the bioink and the support bath together would make it impossible to remove the support bath after printing is completed. For example, transglutaminase can be used as a crosslinker for protein-based bioinks and would pose no problems for a polysaccharide-based support bath (e.g., alginate), but would cause unwanted cross-linking of a protein-based support bath (e.g., gelatin). There is also work being done on the development of universal crosslinkers that can work with a wide range of bioinks and support baths. For example, Hull *et al.* developed a range of UNIversal Orthogonal Network (UNION) bioinks where polysaccharide, protein, and synthetic backbone polymers were used to create bioinks crosslinked by a common click chemistry, where multiple materials can be printed together to form a unified scaffold.^34^ This approach works as long as the UNION crosslinkers can diffuse through the support bath and, in general, as long as this condition is met, nearly any support bath can be used. A list of various cross-linking chemistries compatible with FRESH are listed in Fig. 4.

FRESH is readily performed on a broad range of bioprinting systems capable of motorized or pneumatic syringe-based extrusion and precise 3D motion control within the support bath. Theoretically, this includes but is not limited to pressure-driven deposition heads,^44,49,52^ syringe pump heads,^9,10,24,25,28,31^ microfluidic mixing heads,^21,97–99^ and even pick and place of cells or organoids.^100–102^ Feinberg and co-workers have developed FRESH using custom-designed open-source 3D bioprinters built upon desktop-grade opensource thermoplastic 3D printers.^9,10^ These systems have the advantages of being low-cost (<$2000), small enough to be placed in biosafety cabinets for sterile 3D bioprinting, and easily customizable in terms of both the hardware and software. The instructions and designs for these printers are published in open access journals, the stereolithography (STL) files are distributed through the NIH 3D Print Exchange (https://3dprint.nih.gov/users/awfeinberg/model), and the Feinberg Lab at Carnegie Mellon University also runs an annual workshop where researchers can build their own open-source 3D bioprinter (http://3dbioprint.org/) and has trained more than 30 laboratories. Examples of 3D bioprinters that have been modified from open-source systems include the Lulzbot Mini 1 and 2, FlashForge Creator Pro, PrinterBot Simple Metal, MakerBot Replicator, and MakerGear M2.^9,10,27^ FRESH printing has also been performed on a number of commercial 3D bioprinters including from CELLINK,^43^ Alevi,^44^ Envisiontec,^85^ Aerotech,^52^ Revolution XL,^71^ and RegenHu.^49^ Further, CELLINK and Allevi now directly distribute and support LifeSupport, the commercial version of the FRESH support bath manufactured by FluidForm Inc. This broad hardware compatibility is facilitated by the fact that FRESH uses standard 3D printing software to prepare models for printing and validated printing profiles are available for slicing software such as Slic3r, Cura, and Simplify3D. Importantly, one of the most tedious processes in 3D printing is ensuring that the platform of the printer is perfectly flat and level to the motion control axis. In FRESH, the concept of bed leveling is irrelevant because the extrusion needle can be placed anywhere within the support bath to begin printing. There is no need to find the print bed surface, and even an uneven or unlevel surface would not disrupt the printing process. Further, FRESH printing is performed using a relative coordinate system around X0, Y0, and Z0 origins. This means that to start a print, all you need to do is move the needle to the center of a dish containing the microparticle support bath and click start.

Due to the range of applications and customizability of the FRESH platform, there are some common questions that arise when trying to choose the appropriate conditions such as the optimal yield stress for the support bath, printing speed, nozzle size, and the potential for coalescence of printed material within the support bath. The optimal yield stress for the support bath is dependent on the materials being used, the needle diameter and length, and process parameters such as print speed. For FRESH printing collagen using the gelatin microparticle support bath, we have previously published a range of yield stresses from 1500 to 2500 Pa that we determined as ideal.^10^ Print speed is generally dependent on the intended feature size, printer hardware, and overall print resolution required, with smaller needle diameters requiring slower speeds (∼10 mm/s) but can be higher (∼50 mm/s) for larger needle diameters. Print speed is discussed further in the Future of FRESH section. Another important parameter that affects print resolution, print duration, and feature sizes is the syringe needle diameter used (i.e., nozzle size). Smaller nozzles will allow for higher resolution prints, but with a concomitant increase in the print time. Larger nozzles will significantly decrease the print time due to the increased layer height and filament width but with the trade-off of lower resolution and larger feature size. We have reported printed constructs using nozzles ranging from 20 μm for ultra-high resolution filaments up to 600 μm for rapid printing of larger constructs.^10,27,28,31,32^ Our preferred nozzle size for printing collagen or ECM-based biomaterials is between 80 and 150 *μ*m inner diameters, which balances feature resolution and overall print time. Finally, when bioprinting with FRESH, we have not experienced the coalescence of printed material when using aqueous bioinks in aqueous support baths. We have seen coalescence when there is a hydrophobic/hydrophilic mismatch between the print material and the support bath (e.g., PDMS printed in an aqueous support bath), and so differences in polar and nonpolar solvents and the impact that this has on interfacial energy need to be considered.

## THE FUTURE OF FRESH

**FIG. 5.**
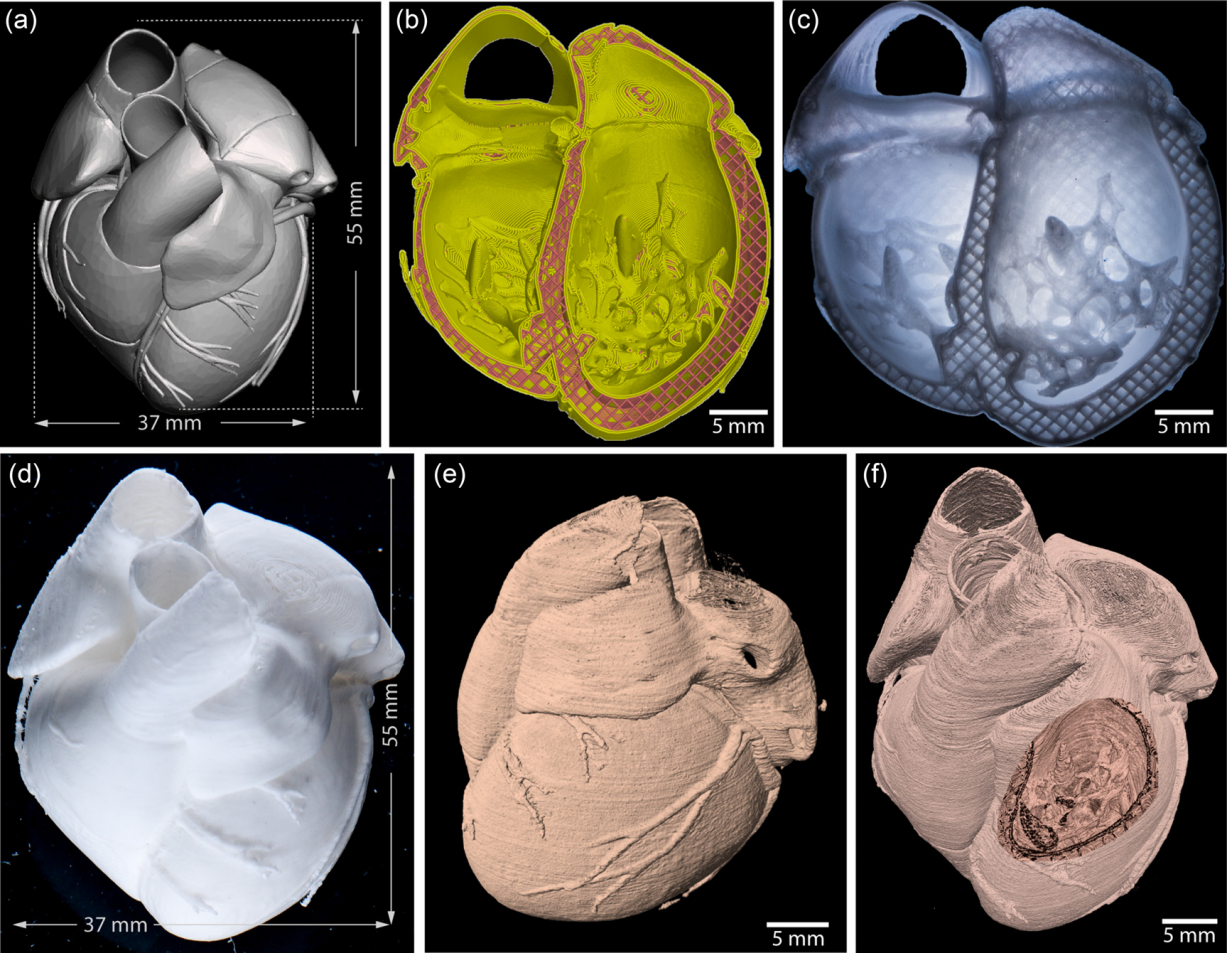
FRESH printing a patient-specific neonatal scale human heart from collagen I. (a) 3D STL model of a human heart scaled to the neonatal size adapted from Lee *et al.*^10^ (b) Visualization of the 3D print pathing for the modeled heart. (c) A half print of the heart revealing the internal complex geometry and the 3D printed infill pattern. (d) A full neonatal scale FRESH-printed heart from collagen I containing barium sulfate for subsequent *μ*CT imaging. (e) A 3D render of the *μ*CT of the FRESH-printed human heart. (f) A windowed view into the interior of the printed heart as viewed by *μ*CT data. These are original unpublished data related to work on collagen bioprinting reproduced with permission from Lee *et al.*, Science **365**(6452), 482–487 (2019). Copyright 2019 American Association for the Advancement of Science.

A major challenge in tissue engineering and regenerative medicine is fabricating functional adult-sized tissues and organs to supplement the limited donor supply for transplant. To date, most 3D bioprinted tissue constructs have been relatively small when compared to the tissues or organs they are intended to replace. In part, this is due to the difficulty in 3D bioprinting soft materials and cells and where FRESH printing and embedded printing have the potential to be a scalable additive manufacturing technology that can produce full-scale tissues based on patient-specific anatomic data. For example, FRESH has been used to create a neonatal-scale physical model of the human heart out of collagen type I based on MRI imaging data.^10^ To do this, the MRI image is segmented to construct a 3D computer model [Fig. 5(a)] and then imported into 3D printing slicing software to generate the machine pathing for the print [Fig. 5(b)]. The construct is then FRESH printed from collagen type I and accurately reproduces the level of details contained in the MRI image, including the internal structure of the ventricles [Fig. 5(c)] and the exterior structure of the heart and vasculature [Fig. 5(d)]. Quantitative assessment of print fidelity is achieved by micro computed tomography (micro-CT) and generation of a 3D model [Fig. 5(e)] and validation of the size and dimensional accuracy, including the complex internal geometry [Fig. 5(f)]. 3D gauging software can also be used to identify regions of overprinting or underprinting during fabrication by comparing the original computer model with the printed physical model.^10,31^ From a fabrication standpoint, the overall size of a FRESH-printed construct is only limited by the build volume of the 3D printer, the dimensions of the support bath container, and the volume of bioink available for extrusion. Production of large volumes of FRESH gelatin microparticle support bath (>10 l) is straightforward using coacervation, and other support baths such as Carbopol are readily available from commercial suppliers in kilogram quantities. While most research-grade 3D bioprinters use syringes that are 10 ml in volume or less, Feinberg and co-workers have previously developed an open-source large volume extruder (LVE) 3D bioprinter capable of extruding up to 60 ml of bioink.^27^ Combined with a large build volume of 15 × 15 × 17 cm^3^, it is possible to FRESH print adult-sized organs such as the heart and kidneys.^32^ In these cases, it is necessary to use a longer, reinforced needle design to prevent unwanted needle deflection in the deeper support bath. There should also be no barrier to printing larger constructs such as the liver or lungs by further increasing the build volume; however, the size is just one of the issues that must be addressed when printing functional tissue constructs.

Being able to print the appropriate bioink for the given application is critically important, and due to the compatibility of the FRESH support bath's aqueous buffer with a wide range of orthogonal gelation mechanisms, it is possible to utilize numerous bioinks within the same printed construct. We have previously implemented this with up to four orthogonal bioinks, FRESH printing collagen type I, alginate, fibrinogen, and MeHA using pH, ionic, enzymatic, and UV light-driven gelation mechanisms, respectively.^10^ Some of these can be expanded upon in a straightforward manner to print an even broader range of materials. For example, bioinks composed of solubilized decellularized ECM can be printed using pH-driven gelation because these are composed predominantly of collagen type I. For photocrosslinkable bioinks, changing the photoinitiator to the one that works at different wavelengths can enable the use of blue, visible, or infrared light instead. If properly selected, it is theoretically possible to use different photocrosslinkable bioinks in the same construct by selecting different wavelengths. Further, other chemistries are also possible in the support bath beyond those already described such as different types of Click chemistry.^34,103,104^ Additionally, it is well known that the composition and concentration of the ECM will directly influence cell behavior.^105–108^ Printing the same bioink such as collagen type I but with different concentrations can be used to tune the mechanical properties of a construct and alter cellular response.^10,107,109^ These are just a few examples that highlight how FRESH provides the ability to use the widest range of bioinks and biomaterials within the same printing environment of any current bioprinting method. However, to take advantage of this, it is necessary to have hardware and software systems that can achieve multimaterial bioprinting. Although it has not yet been implemented with FRESH, a sufficiently advanced tool swapping head (e.g., nScrypt and E3D ToolChanger), or other material switching devices such as a microfluidic head,^21,97–99^ should enable the use of a large number of bioinks in the same tissue construct.

To print larger cellularized tissues and take advantage of large build volumes and multiple materials will require moving beyond the 10 ml syringes found on most 3D bioprinters and even the 60 ml syringe found on our LVE system. There are a number of ways to achieve this, with the simplest possible solution to sequentially refill or load new syringes with newly prepared bioink throughout a print. However, a difficulty for any system that swaps syringes is that the needle tips must be re-aligned in *x*, *y*, and *z* to avoid spatial error in printing following swapping, adding print time. Another option is to move away from syringes and to alternative deposition systems such as a progressive cavity pump that can continuously draw from a master reservoir. While these potential solutions offer ways to increase the total volume of bioink able to be extruded during printing, they do not address settling of bioink mixtures due to gravity. In all cases, a potential problem that one runs into for any long duration print is the possibility of cells settling within the syringe during the printing process.^110–112^ If this occurs, the result is a heterogeneous cell density in the bioink throughout printing, which, in turn, alters the bioink viscosity, making extrusion more difficult, and changes the effective cell concentration. There are several ways to address this challenge, and several research groups have already shown that the addition of rheological modifiers and thickeners like xanthan gum can decrease settling; however, these modifications tend to increase the bioink viscosity creating new problems with printability.^10,110–112^ Ideally, lower viscosity bioinks are preferable as they are easier to extrude and, therefore, subject cells within the bioink to less pressure and shear force.

While increasing the build volume, bioink volume, and bioink types offers advantages in size and complexity of FRESH printed tissue constructs, they do not address a major issue that impacts all 3D bioprinting approaches, which is the overall tissue fabrication time. This is determined by multiple factors, but for extrusion-based approaches, this is largely governed by the print infill density (% solidity of an object) and overall printing speed. Decreasing the percent infill density not only will result in shorter print times but also produces porous constructs that contain void space and often are not desirable when attempting to fabricate a solid tissue. Increasing print speed can shorten the print duration; however, “print speed” is a potentially misleading term in 3D printing. Asking “how fast can something be printed” produces two different interpretations. The first is how quickly can the printer move while printing an object, which, for most extrusion 3D printers, is on the order of 100 mm/s as a maximum. The downside to increasing the movement velocity, especially for embedded printing approaches, is that it can result in lower-quality prints and produce distortion of previously printed layers due to the high-speed stirring of the support bath. The second is how long does it take for an object to be printed from start to finish, which is a function of many factors including % infill density, layer height, movement velocity, and model geometry. All of these can influence the duration of the print, and this ultimately matters in 3D bioprinting of cells because it affects the total amount of time that cells are outside of their ideal incubation environment. While currently, this has not been an issue for the smaller-scale FRESH prints,^10^ as we advance to fabricating large organ-scale constructs, one runs the risk of extending the print time to the point where cellular apoptosis at room temperature could become a concern.

Fabricating larger FRESH printed, cellularized tissue constructs will require achieving long-term viability of cellularized bioinks by regulating metabolic activity through temperature, pH, oxygen, and other methods. While standard CO_2_-independent buffers can be incorporated into most bioinks, heating syringes to 37 °C is needed to maintain physiological conditions. However, cooling syringes to 4 °C may also provide benefit by lowering the cell metabolic rate and, in turn, will slow consumption of the limited nutrients available in the bioink environment. Alternatively, cooling the support bath would offer the same hypothetical benefit as cooling the syringe, namely, slowing cellular metabolism. A key factor to note is that the FRESH gelatin microparticle support bath cannot be raised to 37 °C while printing, as doing so would cause the support bath to melt prematurely and no longer provide support to the printed components. In this case, instead of gelatin-based FRESH support baths, other temperature insensitive support baths may be used such as alginate or agarose-based baths that do not liquefy at 37 °C. In these cases, the challenge becomes removing the support bath later while maintaining high cell viability. For very long duration prints, it may be beneficial to treat the print container as an incubator to further extend cell viability rather than relying on cooling to slow cell metabolism or warming of the bioink within the syringe. Finally, it may also be possible to manipulate the cells directly to alter their metabolic function through small molecules or other chemicals, although this has yet to be explored in the literature.

The advanced printing capabilities of FRESH, while allowing for complex geometries, also introduce difficulties in ensuring that the final geometry is accurate. Until recently, the majority of bioprinted constructs were simple prismatic shapes or even simpler stacked square lattices. Furthermore, due to the limitations of printing in air, they were relatively thin. These restrictions lend themselves to assessing print fidelity by simple light microscopy or other visual inspection methods. While printing complex internal geometries within thick constructs, these approaches become unusable. Instead, the print fidelity must be assessed during printing by in-process monitoring. Depending on the implementation, multiple imaging modalities could be utilized. If fluorescent bioinks were used, a confocal or multiphoton microscope could be integrated into a robotic print system, capturing images as the construct is built. Other volumetric imaging modalities, such as optical coherence tomography and computed tomography, could also be implemented. With in-process imaging, it would be possible to continuously assess the fidelity of the print, using the machine pathing as a reference. Additionally, during multi-material printing, imaging would allow for verification of needle alignment, which is extremely important and becomes increasingly challenging with more materials. Furthermore, anomalies such as clogged needles, trapped air bubbles, and debris within the construct could be identified during the print, and the printing process could be paused or terminated with an indication for the user to save valuable reagents and bioinks. Finally, after a successful print, a full volumetric model of the printed construct could be assembled from sequential in-process images and compared in a number of ways with the digital prototype model (which was initially used to generate the machine pathing). Deviations from intended geometry can be assessed using 3D gauging software already developed for subtractive machining.^10^ Depending on the thickness and dimensions of the construct, this in-process image acquisition might be the only viable method of capturing internal details.

Finally, the majority of current 3D bioprinting techniques, including most implementations of FRESH, utilize a layer-by-layer printing approach. However, since the support bath minimizes the effect of gravity, FRESH allows for printing with simultaneous motion of more than two axes. For example, a helix can be printed directly, with a trajectory that simultaneously moves in the X, Y, and Z axes of the printer (Fig. 4).^9^ With more axes of motion, it becomes possible to orient the needle to be orthogonal to the filament trajectory, allowing for much smoother, optimized nonplanar, or lattice constructs to be printed. Additionally, one can imagine a dynamic print container that provides access from the top and sides of a construct to allow for even greater freedom of printing. However, with this approach, issues can arise where the needle interacts with previously printed filaments in 3D space. Thus, while FRESH itself will allow for true freeform printing, easily available software and print planning tools for such sophisticated computer-aided manufacturing (CAM) motion have not yet been developed for 3D bioprinting although five axis machining CAD/CAM packages could likely be modified or adapted to speed this advancement. To date, FRESH printing utilizes commercially available or open-source slicing software to generate print pathing designed for plastic printing. With some simple modifications to parameters such as filament diameter and nozzle diameter, these programs are well suited for bioprinting. However, as more sophisticated implementations of FRESH become necessary, such as nonplanar or greater than three axis simultaneous motion, these programs will no longer be sufficient. Instead, it is likely that a new class of print pathing programs will need to be developed that are similar to subtractive machining CAD/CAM packages. Sophisticated motion control for additive manufacturing processes is already being developed by numerous machine tool companies and their partners (e.g., DMG Mori LASERTEC line). These tools are generally limited to metal printing but would be adaptable to bioprinting. Working with these companies or perhaps mimicking their software in open-source implementations would allow bioprinting with FRESH to reach new levels of 3D complexity.

## CONCLUSION

In summary, FRESH is a customizable approach enabling the fabrication of biological and synthetic constructs from soft materials via embedded printing within a yield-stress support bath. By pairing specific microparticle support baths with an aqueous buffer to support bioink specific gelation mechanisms, FRESH supports a broad range of print materials and cells for advanced biofabrication. Since the emergence of FRESH and related embedded printing techniques in 2015, the field has seen rapid adoption and growth, including adoption by dozens of laboratories worldwide and commercial availability from major bioink and bioprinter manufacturers. This has produced a range of research advances including high-resolution ECM-based scaffolds of organ models from patient-specific medical imaging datasets and the creation of beating cardiac tissue.^9^ While significant work has been done, thus far, to expand the capabilities and applications of embedded bioprinting, we believe that we are only at the beginning of what techniques such as FRESH can offer to tissue engineering and 3D biofabrication.

## Data Availability

Data sharing is not applicable to this article as no new data were created or analyzed in this study.
